# Cardiac arrhythmia and neuroexcitability gene variants in resected brain tissue from patients with sudden unexpected death in epilepsy (SUDEP)

**DOI:** 10.1038/s41525-018-0048-5

**Published:** 2018-03-27

**Authors:** Daniel Friedman, Kasthuri Kannan, Arline Faustin, Seema Shroff, Cheddhi Thomas, Adriana Heguy, Jonathan Serrano, Matija Snuderl, Orrin Devinsky

**Affiliations:** 1 Comprehensive Epilepsy Center, NYU Langone Medical Center, New York, NY USA; 2 Department of Neurology, NYU Langone Medical Center, New York, NY USA; 3 Department of Pathology and Genome Technology Center, NYU Langone Medical Center, New York, NY USA

## Abstract

Sudden unexpected death in epilepsy (SUDEP) is the leading cause of epilepsy-related mortality in young adults. The exact mechanisms are unknown but death often follows a generalized tonic–clonic seizure. Proposed mechanisms include seizure-related respiratory, cardiac, autonomic, and arousal dysfunction. Genetic drivers underlying SUDEP risk are largely unknown. To identify potential SUDEP risk genes, we compared whole-exome sequences (WES) derived from formalin-fixed paraffin embedded surgical brain specimens of eight epilepsy patients who died from SUDEP with seven living controls matched for age at surgery, sex, year of surgery and lobe of resection. We compared identified variants from both groups filtering known polymorphisms from publicly available data as well as scanned for epilepsy and candidate SUDEP genes. In the SUDEP cohort, we identified mutually exclusive variants in genes involved in µ-opiod signaling, gamma-aminobutyric acid (GABA) and glutamate-mediated synaptic signaling, including *ARRB2*, *ITPR1*, *GABRR2*, *SSTR5*, *GRIK1*, *CTNAP2*, *GRM8*, *GNAI2* and *GRIK5*. In SUDEP patients we also identified variants in genes associated with cardiac arrhythmia, including *KCNMB1*, *KCNIP1*, *DPP6*, *JUP*, *F2*, and *TUBA3D*, which were not present in living epilepsy controls. Our data shows that genomic analysis of brain tissue resected for seizure control can identify potential genetic biomarkers of SUDEP risk.

## Epilepsy: Spotting genetic drivers of sudden death

Gene variants associated with abnormal heart rhythm and neuronal excitability may increase the risk of Sudden Unexpected Death in Epilepsy (SUDEP). SUDEP is the most common cause of death directly related to epilepsy, but little is known about the risk factors and mechanisms through which seizures can lead to death. Daniel Friedman, Orrin Devinsky and colleagues at New York University Langone Medical Center, US, compared whole-exome sequences from brain tissue belonging to eight epilepsy patients who died from SUDEP and seven matched living controls who had brain tissue removed for epilepsy treatment. In the SUDEP cases they identified 13 rare gene variants involved in cardiac arrhythmia and excitatory neurotransmission as potential genetic biomarkers of SUDEP risk. Further understanding the genetic contribution to epilepsy-related mortality will help develop effective preventive strategies.

## Introduction

Epilepsy is a neurologic disorder, with a prevalence of ~1.2% in the United States.^1^ People with epilepsy (PWE) experience increased mortality compared to the general population including a 24-fold increased risk of sudden death.^2^ Sudden unexpected death in epilepsy (SUDEP) is a death in a previously well person with epilepsy that does not result from trauma, drowning, status epilepticus and in whom the post-mortem examination does not reveal an alternative cause of death.^3^ SUDEP is perhaps the leading cause of epilepsy-related death with an annual incidence of 1 per 1000 patients. However, this rate is much higher among patients with difficult to control seizures and occurs in ~1/150 PWE per year among epilepsy surgery candidates.^4^ SUDEP is typically unwitnessed but cases observed by family or caretakers, or recorded in epilepsy monitoring units, reveal that generalized tonic–clonic seizures (GTCS) usually precede death. The mechanisms of SUDEP remain incompletely defined; animal studies, epidemiology, physiology and pathology suggest contributions from neurobiological (e.g., duration of postictal EEG suppression, a likely marker of profound brain dysfunction or ‘shutoff’) and environmental (e.g., positional asphyxia and lack of nocturnal supervision by an individual who can aid the patient after a seizure) factors.^5^

The genetic contribution to SUDEP risk is poorly understood. Several studies support a role for genetic factors associated with early onset, treatment-resistant epileptic encephalopathies such as mutations in S*CN1A* (Dravet syndrome),^6^ mutations in *SCN8A*,^7^ and Dup15q11 syndromes^8^ as well as treatment-resistant focal epilepsies such as *DEPDC5*.^9^ It remains uncertain if these genes are associated with SUDEP solely due the risk of severe epilepsy or if other features are involved. For example, impaired brainstem or cardiac sodium channel function in patients with sodium channel gene mutations or impaired GABRB3 receptor function in Dup15q patients may increase SUDEP risk independent of epilepsy severity. Evidence from animal models and human case studies suggest that genetic variants associated with cardiac arrhythmias may contribute to some cases of SUDEP. For instance, *KCNH2* mutations can increase the risk of long QT syndrome (LQT) and epilepsy^10^ and known or suspected pathological *KCNH2* variants are more frequent among decedents with SUDEP.^11,12^ Since RNA sequencing data reveal that most ion channel genes are expressed in brain and heart, albeit to markedly different degrees (e.g., *SCN1A* more in brain; *SCN5A* more in heart), mutations in a single gene can alter excitability in both myocardium (e.g., pacemaker, conduction, myocardium) and brain (e.g., cortex, brainstem). Recently, definite pathogenic or candidate pathogenic variants, in genes associated with cardiac arrhythmia or epilepsy, were identified in 28 of 61 (46%) of SUDEP cases.^11^

Genetic studies of SUDEP have focused on germline variants to identify candidate mutations in DNA derived from peripheral blood. However, the mutational landscape of brain tissue and particularly of epileptogenic cortex, remains unknown. In striking contrast to precision medicine in cancer, pathologic brain tissue removed for epilepsy treatment does not routinely undergo molecular genetic analysis as part of the clinical neuropathology analysis. To elucidate mutational burden we performed a comprehensive genomic analysis of resected epileptogenic cortex. We also tested the feasibility of the whole-exome sequencing (WES) and variant analysis of the formalin-fixed paraffin-embedded (FFPE) epileptogenic brain tissue. While the lack of blood germline, which could help identify somatic brain mutations, represents a limitation, of our study, FFPE brain tissue is the most common material available to be studied in the clinical surgical neuropathology setting. Therefore, developing molecular methods and bioinformatic pipelines to analyze FFPE brain seizure samples could potentially inform clinical management, similar to current cancer therapy. Here we report WES of epileptogenic cortex from SUDEP patients and compare their mutational profile with tissue from matched controls of patients who underwent surgery but did not die of SUDEP.

## Results

### Case selection and WES analysis

**Fig. 1:**
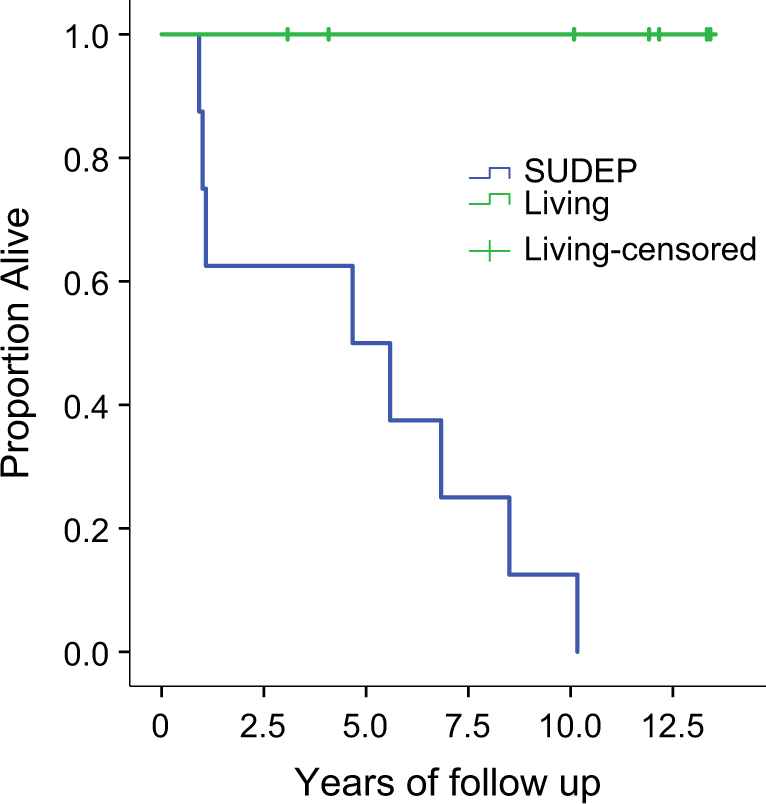
Survival analysis of SUDEP and living epilepsy patients control group. Patients were matched for age of onset of seizure and age of surgery

Eight patients had FFPE neuropathological surgical specimens available for study (SUDEP patients, Table 1). These were matched with specimens from seven patients who had surgery and were alive at the time of analysis (termed “living epilepsy”, Table 1). Both SUDEP and living epilepsy groups were matched for age at surgery (median 37 and 34 years, respectively) and age of seizure onset (median 13 and 10 years, respectively). None of the SUDEP patients and only two of the living epilepsy patients (Pts. #2 and #5) were seizure-free after surgery. None of the SUDEP or living epilepsy patients had a personal history of cardiac arrhythmia; detailed family history was unavailable for most of the patients in the medical records. Median survival from surgery to death was 5.5 years in SUDEP patients (range, 1–11 years) and mean duration of follow up was 9.7 years in living epilepsy patients (range, 3–12 years). Survival was significantly longer for the living epilepsy group (Mantel–Cox* χ*^2^ = 11.9, *p* = 0.001; Fig. 1). Histological evaluation of resected epileptic tissue was performed by three neuropathologists (MS, SS, AF) and the most common findings were mild architectural abnormalities of the cortex corresponding to focal cortical dysplasia (FCD, see Table 1 for subtypes). There were no histological differences in the type or extent of FCD subtypes according to International League Against Epilepsy classification scheme^13^ in SUDEP versus living epilepsy patients (Table 1). DNA was extracted and WES performed from FFPE tissue of the dysplastic cortex. As these were archival surgical tissue samples, germline (blood, saliva) DNA was unavailable for analysis.

**Table 1:** Clinical characteristics of SUDEP cases and matched living epilepsy patients

Case #	Age	Sex	Race	Age of onset (years)	Seizure	PMH	Lobe of surgery	Pathology (ILAE class)	Age of death /last follow-up
SUDEP patients									
1	37	F	WNH	12	CPS, SGTC	Postictal psychosis	R Temp	FCD IA	44
3	41	M	WNH	23	CPS, SGTC	None	L Temp	FCD IIA	47
5	37	M	WNH	<1	CPS, SGTC	Depression, anxiety	L Temp	FCD IIIA	45
7	23	M	WH	<1	CPS, SGTC	None	R Front	FCD IA	25
9	34	M	WH	15	SPS, CPS	Depression, postictal psychosis	R Temp	FCD IA	36
11	51	M	WH	47	SPS, CPS, SGTC	Alcohol abuse	R Temp	FCD IA	52
13	43	M	WNH	NA	NA	NA	R Temp	FCD IA	54
16	21	F	WNH	13	SPS, CPS, SGTC	None	R Temp	FCD IB	26
Living epilepsy patients									
1	37	F	WNH	31	SPS, SGTC	Headaches	L Temp	FCD IA	49
2	34	M	WH	4	CPS	Depression, anxiety	R Temp	FCD IIIA	35
3	54	M	WNH	10	CPS, rare SGTC	None	L Temp	FCD IB	62
4	45	M	WNH	10	CPS	Hyperlipidemia	R Temp	FCD IIIA	56
5	20	F	WH	5	CPS, SGTC	Postictal psychosis	L Temp	FCD IA	32
6	32	M	WNH	19	CPS	Hydrocephalus, developmental delay	R Temp	FCD IIA	35
7	16	F	WNH	5	SPS, CPS, SGTC	ADHD	L Temp	FCD IA	28

*PMH* past medical history, *ILAE class* International League Against Epilepsy Classification of Corticla Dysplasia Type, *Temp* temporal lobe, *WH* white, Hispanic, *WNH* white, non-hispanic, *SPS* simple partial seizures, *CPS* complex partial seizures, *SGTC* secondarily generalized tonic–clonic seizures, *FCD* focal cortical dysplasia, *ADHD* attention deficit-hyperactivity disorder, *NA* not available

**Fig. 2:**
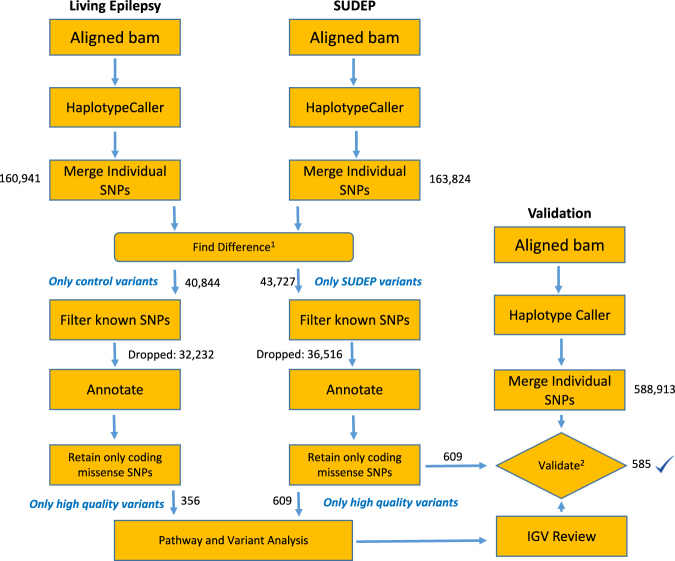
Workflow of the comprehensive analysis of archival brain tissue whole-exome seqencing in the absence of the matched germline DNA. Identification of variants is performed by pathway analysis and pathogenicity analysis is performed following the ACMG/AMP guidelines. Find difference (1) process removes variants common to both living epilepsy and SUDEP tissue. The validation process (2) involves replication of all the steps from DNA extraction to whole-exome sequencing from a second section of seizure focus tissue from the same patient. Only variants found in both samples were included in subsequent analysis. SNPs single-nucleotide polymorphisms, IGV integrated genomics viewer, ACMG American College of Medical Genetics and Genomics, AMP Association for Molecular Pathology

The total number of SNPs detected in WES per case in the SUDEP group ranged from 72177 to 79523, mean, 74140; and in the living epilepsy group ranged from 62716 to 76736, mean 71634 case. High frequency SNPs found in the following public, databases: 1000 g, ESP6500, gnomAD and dbSNP141, were filtered out (Fig. 2).

### Genetic landscape of epileptogenic brain foci in SUDEP and living epilepsy patients

The mean fold coverage was 111.9 ± 16.5 for SUDEP and 66.5 ± 35 for living epilepsy patients’ brain samples. We identified 662 rare variants in SUDEP seizure foci and 386 rare variants in seizure foci of living epilepsy patients (Fig. 2 and Supplemental Tables 2 and 3, Supplemental Fig. 2). Identified variants were compared to a list of manually curated 584 epilepsy and candidate SUDEP genes (Epilepsy/SUDEP Genes, Supplemental Table 4) showing relatively little overlap among the three categories highlighting the heterogeneity of this disease (Supplemental Fig. 1).

**Fig. 3:**
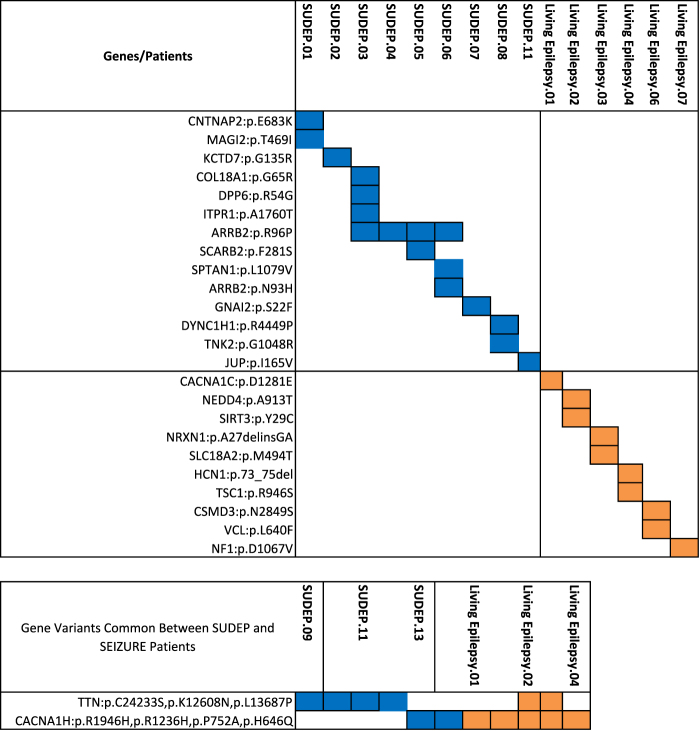
Mutational landscape of curated genes associated with epilepsy or SUDEP in tissue from SUDEP (blue) and living epilepsy (orange) patients. Variants in these curated genes that were unique to a group are shown in the top table whereas variants found in both groups are shown in the table below

We identified 14 potentially pathogenic variants in 13 genes in eight SUDEP patients and ten potentially pathogenic variants in ten genes in six living epilepsy patients present in Epilepsy/SUDEP gene list (Fig. 3). No subjects had known pathogenic variants in these genes. Two SUDEP patients and one living epilepsy patient had rare variants in *TTN* (codes for a large protein in cardiac and skeletal muscle; disorders including cardiomyopathy and myopathy with early respiratory failure; OMIM: 188840) and CACNA1H (calcium channel, voltage-dependent, T Type, alpha 1 H subunit; disorders include tonic–clonic and febrile seizures; OMIM: 607904) though the loci were unique. Four SUDEP subjects had variants in *ARRB2* (arrestin, beta 2, OMIM# 107941); all four subjects shared the same rare variant (gnomAD total allele frequency <0.05%) and one subject also had another variant in the same gene. Polymorphisms in ARRB2 have been associated with µ-opiod receptor sensitivity^14^ and perceived breathlessness in patients receiving opiods.^15^ Additional potentially pathogic variants and variants of unknown signficance (VUS) occurred in genes associated with epilepsy/SUDEP that were unique to individual subjects. Additional potentially pathogenic variants and VUS in curated epilepsy/SUDEP genes in SUDEP brain tissue included *COL18A1* (Collagen, type XVIII, alpha-1, associated with Knobloch syndrome type 1, OMIM# 120328), *JUP* (junction plakoglobin, arrythmogenic right ventricular dysplasia 12 and Naxos syndrome, OMIM#173325), *DPP6* (dipeptidyl peptidase VI, associated with paroxysmal ventricular fibrillation, OMIM# 126141), *KCTD7* (Potassium channel tetramerization doman containing protein 7, associated with progressive myoclonic epilepsy 3, OMIM# 611725), *GNAI2* (guanine nucleotide-binding protein, alpha-inhibiting acitivity polypeptide 2, associated with idiopathic autosomal dominant ventricular tachycardia, OMIM#139360), *CNTNAP2* (contactin-associated protein-like 2, associated with FCD syndrome, OMIM# 604569), *SCARB2* (scavenger receptor class B, member 2, associated with progressive myoclonic epilepsy, type 4, OMIM# 602257), and *SPTAN1* (alpha spectrin, nonerythrocytic 1, associated with early infantile epileptic encephalopathy, OMIM# 182810).

Genes with potentially pathogenic variants in living epilepsy brain tissue included *CACNA1C* (calcium channel, voltage-dependentdependennt, T-type, alpha 1 C subunit, associated with Timothy and Brugada syndromes, OMIM# 114205), *HCN1* (hyperpolarization-activated cyclic nucleotide-gated potassium channel, associated with early infantile epileptic encephalopathy, OMIM#602780), *TSC1* (hammartin, associated with tuberosis sclerosis complex, OMIM# 605284), *VCL* (vinculin, associated with dilated and hypertrophic cardiomyopathy OMIM#193065), *CSMD3* (cub and sushi multiple domains 3, neurodevelopmental disorders and familial benign myoclonic epilepsy, OMIM# 608399) and *NRXN1* (neurexin I, neurodevelopmental disorders, OMIM # 600565). A full list of potentially pathogenic and VUS in living epilepsy and SUDEP brain tissue are found in Fig. 3 and Supplemental Tables 2 and 3.

**Fig. 4:**
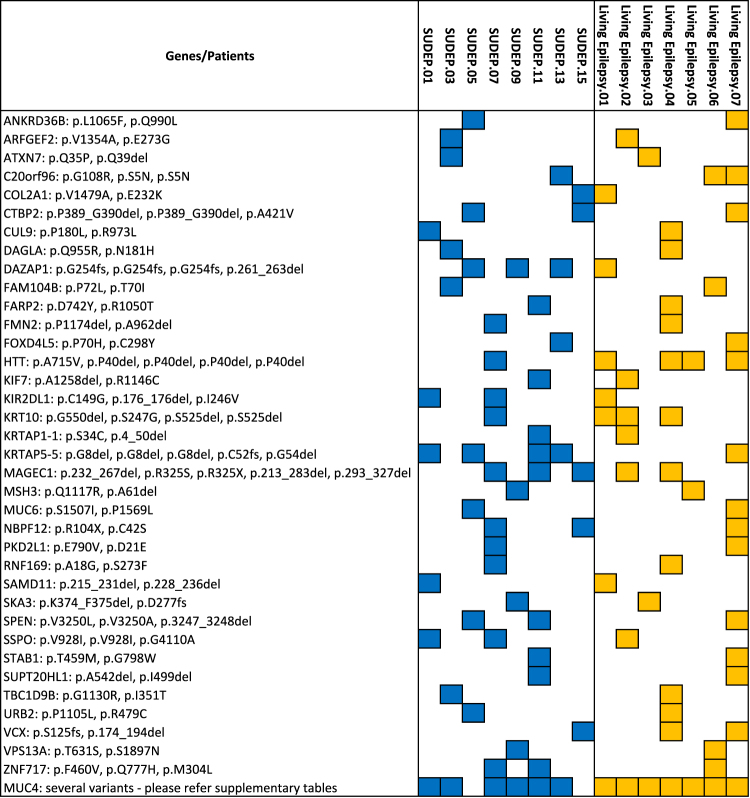
Novel variants in tissue from both in SUDEP (blue) and living epilepsy (orange) patients

SUDEP and living epilepsy patients shared potentially pathogenic variants in 37 genes not previously implicated in epilepsy (Fig. 4). The potential pathogenic role of these variants remains to be elucidated; however, a query of gene expression database revealed that 30 of 36 genes for which human data exist were expressed in the brain or heart. The vast majority of all rare variants were unique to either SUDEP (*n* = 610) or living epilepsy (*n* = 337) patients (Supplemental Fig. 1).

### Pathway analysis identifies critical role of GABA/glutamate pathway and cardiac transduction in SUDEP patients

**Fig. 5:**
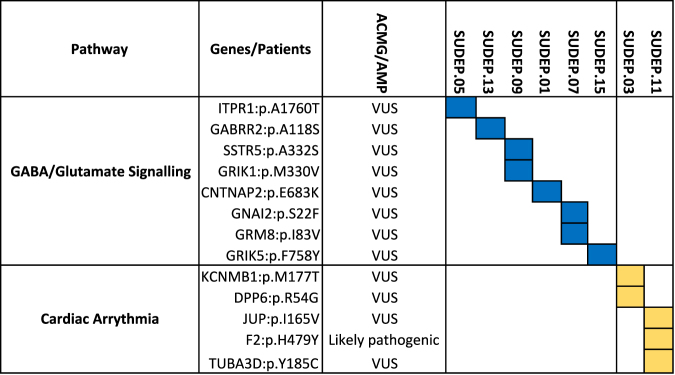
Variants in GABA/glutamate signaling and cardiac transduction genes in SUDEP patients. Following the ACMG/AMP guidelines, most novel variants are identified as variants of unknown significance (VUS). Variant in F2 is identified as likely pathogenic

Next we investigated what pathways were unique to SUDEP patients based on their mutational profile. These variants were analyzed using Ingenuity™ Pathway (IPA) and Variant Analysis (IVA; ingenuity.com) to identified mutually exclusive rare variants in cardiac arrhythmia and neuroexcitability pathways (Fig. 5). Allele frequency of these variants was ≤ 0.0022 (mean = 1.8 × 10^–4^) in the gnomAD database (Supplmental Table 9). We found variants in genes involved in gamma-aminobutyric acid (GABA) and glutamate-mediated synaptic signaling, including *ITPR1, GABRR2, SSTR5, GRIK1, CTNAP2, GRM8, GNAI2 and GRIK5*. We also identified five potentially pathologic variants in genes associated with cardiac arrhythmia, including *KCNMB1, KCNIP1, DPP6, JUP, F2, and TUBA3D* that were identified in SUDEP patients but not present in our living epilepsy cases. (Fig. 5). When these variants were analyzed following the ACMG standards and guidelines,^16^ none were identified as benign and all but one were identified as variants of unknown significance (VUS). The *F2* p. H479Y mutation was identified as likely pathogenic (full analysis with ACMG scores for each category available in Supplemental Table 5).

Some of these genes are associated with human disease: *KCNMB1* - resistance to diastolic hypertension (OMIM: 608622)^17^; *DPP6* - paroxysmal familial ventricular fibrillation type 2 and idiopathic ventricular fibrillation (OMIM: 126141)^18^; *JUP* - Naxos disease, a recessively inherited condition with arrhythmogenic right ventricular dysplasia/cardiomyopathy (ARVD/C) and a cutaneous abnormalities (OMIM: 173325).^19,20^ We found no reports implicating *TUBA3C/TUBA3D* (OMIM: 602528) variants in arrythmogenic sudden-death risk.

Potential pathogenic variants identified in SUDEP but not living epilepsy cases in GABA/glutamate signaling pathways included *CNTNAP2* (OMIM: 604569), which is associated with FCD–epilepsy syndrome^21^ and *ITPR1* (OMIM: 147265), which is associated with spinocerebellar ataxia SCA15^22^ and seizures in mice lacking this gene.^23^ We identified variants in genes linked to epilepsy in GWAS studies, including *GRM8* (OMIM: 601116), photoparoxysmal response on EEG^24^; *GABRR2* (OMIM: 137162), sporadic epilepsy^25^; and *GRIK1* (OMIM: 138245), juvenile absence epilepsy.^26^ The role of variants *SSTR5* (OMIM: 182455) and *GRIK5* (OMIM: 600283) in epilepsy risk were not previously reported. All of these variants were determined to be VUS (see Supplemental Table 5).

We also identified a VUS in *GNAI2* (OMIM: 139360) in one patient. This gene is associated with glutamate/GABA signaling as well as cardiac arrhythmias, including idiopathic ventricular tachycardia.^27^

## Discussion

Our study supports the feasibility of extracting and sequencing DNA of sufficient quality for next-generation sequencing whole exome analysis from epilepsy surgical specimens up to 15 years post-resection, to identify potential pathogenic variants in the brain tissue without available germline DNA, using comprehensive bioinformatics analyses. Our small cohort (eight SUDEP, seven living epilepsy) identified previously unreported gene variants in epilepsy patients, not present in currently available germline exome/genome SNP databases or living epilepsy surgical patients. We identified potentially pathogenic variants in genes associated with epilepsy or neurodevelopmental delay in six of eight SUDEP brain specimens and five of seven living epilepsy specimens. Potentially pathogenic variants associated with cardiac arrhythmia were seen in three SUDEP cases and two living epilepsy cases. Four of the eight SUDEP specimens had the same potentially pathogenic nonsynomous SNP in ARRB2, with one having a second variant in the same gene. Pathway analysis revealed novel variants in two major signaling pathways potentially relevant to SUDEP and epilepsy. Five SUDEP cases had variants in genes regulating excitatory and inhibitory synaptic transmission and two patients had variants in genes potentially involved in cardiac arrhythmias. One had a variant in a gene involved in both neuroexcitability and cardiac pathways. All variants are distinct from those previously associated with SUDEP cases in molecular autopsy series.^11,12^ Prior observation on mutations in specific genes in SUDEP cases (e.g., *KCNH2, DEPDC5, LQT1, LQT2, LQT3 and ANK2*) were not observed in our SUDEP cases or living epilepsy controls. This likely reflects our small sample size and that SUDEP is a heterogeneous condition and multiple genes and variants may contribute to risk.

There were likely pathogenic or pathogenic variants in 610 genes that were identified in the entire SUDEP group compared to 337 in the living epilepsy group. Although the median number of pathogenic and likely pathogenic variants per subject were higher in the living epilepsy group compared to the SUDEP group (18 vs. 12), the nearly equal number of VUS (1556 vs. 1589) suggests that SUDEP could be driven by novel mutations rather than known epilepsy pathogenic drivers.

While we show that WES analysis can be performed on archival FFPE brain tissue material, there are limitations to this study. The small sample size precludes any definitive conclusions regarding the relationship between unique gene variants in SUDEP cases and SUDEP risk. DNA used in analysis was extracted from archival epileptogenic tissue and no blood samples were available for comparison. Therefore, it is unknown if the identified variants were somatic or germline mutations nor the extent of the distribution of the genetic variants throughout the brain, heart or other other tissues. This limitation is inherent to studies that focus exclusively on DNA derived from blood and are blind to potential somatic mutations in brain or heart. For cardiac arrhythmia gene variants, our brain-derived DNA makes it more uncertain if these variants were present in the heart and contributed to arrhythmia risk.

Another limitation of our study is lack of functional characterization of the identified novel variants. We did not identify known pathological variants in our analysis of epilepsy and SUDEP-related genes or through pathway analysis so the relationship of these variants to SUDEP or epilepsy remains speculative and requires further testing in future studies. However, half of the SUDEP cases shared the same variant in the *ARRB2* gene, which encodes beta-2-arrestin, a protein that is involved in modulating desensitation response in G-protein-coupled receptors. Studies in animal models have shown that beta-2-arrestin is critical to densensitization of µ-opoid receptors and mice lacking this receptor have enhanced effects of exogenous opiods.^28^ Pharmacogenomic studies have shown that polymorphisms in the *ARRB2* gene are associated with treatment response in patients treated with opiod analgesics for refractory dyspnea.^15^ Central hypoventilation and apnea, perhaps related to seizure-induced release of endogenous opiods, is thought to be a mechanism for SUDEP and a randomized clinical trial of the opiod receptor antagonist naltrexone to prevent postictal respiratory dysfunction and coma is currently in progress.^29^ Epilepsy patients with polymorphisms which impair desensentization of brainstem opiod receptors in response to seizure-related endogenous opiod release may experience more severe postictal apnea, thereby increasing SUDEP risk. Further studies in larger samples are needed to confirm the association of this gene with SUDEP risk.

Using pathway analysis, we identified three previously unreported variants unique to SUDEP cases in cardiac conduction pathway genes. Other SUDEP molecular autopsy series identified known and suspected pathogenic variant in genes associated with lethal arrhythmia risk, such as LQT2 and Brugada syndrome.^11,12,30^ While these genes may increase SUDEP risk by lowering the threshold for cardiac arrhythmias during the fatal postictal cascade, cardiac arrhythmia was not observed in any of the ten SUDEP cases with concurrent EEG and ECG monitoring.^31^ The contribution of these variants to SUDEP risk in the general population remains unclear. However, the role of these genetic variants in SUDEP may be mediated by brainstem rather than cardiac effects.^5^

We also identified six previously unreported variants, unique to SUDEP cases, in glutamateric and GABAergic neurotransmission. These variants could influence excitatory:inhibitory (E:I) balance and increase risk of epilepsy, seizure severity or centrally mediated autonomic dysfunction. Impaired inhibitory neurotransmission or excessive excitatory neurotransmission can enhance focal seizure spread and secondarily generalization by increaseing E:I balance in cortex outside the seizure-onset zone. Frequent GTCs are the greatest clinical risk factor for SUDEP.^32^ Effects on E:I balance could also disrupt postictal neuronal recovery. SUDEP usually occurs during the postical period with prolonged depression of arousal and respiratory reflexes. Aberrations in synaptic signaling could influence brainstem networks that impact excitability and predispose to spreading depression which is associated with SUDEP in mouse models^33,34^ or to seizure invasion of brainstem structures.^35^

Studies have not revealed definite genetic risk factors for SUDEP. Molecular autopsies of SUDEP cases suggest some decedents carry mutations and potentially pathogenic variants in genes associated with cardiac arrhythmias such as *KCNQ1* as well as genes associated with severe epilepsies such as SCN1A and DEPDC5.^11,12,30,36^ However, these variants occur in a minority of SUDEP cases and additional genetic determinants of SUDEP risk likely exist but remain elusive due to the relatively low frequency of SUDEP, heterogenous mechanisms of seizure-related death, and potentially diverse genetic factors. Further, some SUDEP mechanisms may reflect acquired and not genetically-determined mechanisms. For instance, serotonergic and respiratory systems are implicated in SUDEP and other sudden death syndromes such as sudden infant death syndrome (SIDS).^37^ However, no study that we are aware of, including ours, has uncovered variants in genes or promoters involved with serotonergic signaling or respiratory function associated with SUDEP or SIDS.^38^

Our study shows feasibility of WES from the surgical epilepsy specimens and identification of novel variants without germline DNA using comprehensive bioinformatics approaches. Without DNA derived from blood or saliva, FFPE brain tissue can provide relevant insight in the genetics of the disease although matched blood samples should be obtained when possible to identify somatic brain variants. While preliminary, our findings are concordant with the largest published study of SUDEP genomics, and implicate new genes involved in opiod receptor function, neural excitability and cardiac function as potential SUDEP mechanisms. Acording to the ACMG guidelines all but one were annotated as VUS, suggesting a potential association with SUDEP. Future studies need to assess genetic findings in both high risk (e.g., epileptic encephalopathies and treatment-resistant focal epilepsies) and low risk (e.g., epilepsies of recent onset or infrequent seizures), compare the germline and somatic variants in SUDEP and other epilepsy surgery cases, and develop precision medicine approaches to predict SUDEP risk. Further, correlating specific clinical features of the patients’ epilepsy and SUDEP setting/mechanisms may improve our understanding of how genetic variants contribute to SUDEP risk.

## Methods

### Patient samples and pathology analysis

The study was approved by the NYU Institutional Review Board. We reviewed the NYU epilepsy surgery clinical database who had surgery between 1999–2013 and and identified ten patients who died from definite/probable SUDEP using the definitions of Nashef and colleagues.^3^ All deaths were adjudicated by two epileptologists with expertise in SUDEP (DF and OD). Eight had formalin-fixed paraffin embedded brain tissue available from epilepsy surgery for DNA extraction. Seven control samples from patients who had undergone epilepsy surgery matched for age at surgery, sex, year of surgery and lobe of resection (Table 1). Hematoxylin & eosin sections were reviewed and cortical dysplasias classified according to the international consensus criteria^13^ by three neuropathologists (MS, SS and AF). These foci with cortical dysplasia were selected for WES.

### WES and bioinformatics analysis

For each case in our study, DNA was extracted from ten unstained FFPE sections, which were cut at 10 µm thickness. DNA was extracted using automated DNA extraction Maxwell RSC DNA FFPE (Promega, Madison, WI) extraction kit following CLIA validated clinical protocol. 250 ng of DNA from each sample were sheared on a Covaris instrument for 360 seconds (duty cycle—10%; intensity—5; cycles/burst—200). Barcoded libraries were prepared using the Kapa Low-Throughput Library Preparation Kit Standard (Kapa Biosystems), amplified using the KAPA HiFi Library Amplification kit (Kapa Biosystems) (eight cycles) and quantified using Qubit Fluorimetric Quantitation (Invitrogen) and Agilent Bioanalyzer. An equimolar pool of the four barcoded libraries (300 ng each) were used as input to capture the exome using one reaction tube of the Nimblegen SeqCap EZ Human Exome Library v3.0 (Roche, cat # 06465684001), according to the manufacturer’s protocol. The pooled capture library was quantified by Qubit (Invitrogen) and Bioanalyzer (Agilent) and sequenced on an Illumina HiSeq 2500 using a paired end, 100 nucleotides in length run mode. The lower coverage of living epilepsy cohort is attributed to the lower DNA quality as determined by the PCR duplication rate after sequencing and alignment (16% duplicates vs. 8% for SUDEP cohort; both normal level of duplicates for FFPE DNA in WES). For SNP identification, the coverage was normalized by HaplotypeCaller (GATK) when calculating the quality scores of the variants and the variants were called independently for each sample with an adequate coverage of 65. In general, most variants can be called with 30× coverage, which is routine for germline variants therefore this difference in coverage between SUDEP and living epilepsy WES is irrelevant for our findings. Sequencing statistics are summarized in the Supplemental Table 1. Realigned exomes were queried for SNPs using HaplotypeCaller. High frequency SNPs found in 1000 g, ESP6500, gnomAD and dbSNP141 were filtered out. Resulting filtered variants were annotated using ANNOVAR RefSeq hg19. Synonymous variants were excluded. Variants were grouped by genes and analyzed using MSigDB, IPA, Reactome and CarpeDB databases. Ingenuity™ pathway (IPA) and variant analysis (IVA; ingenuity.com) was performed to identify candidate mutations involved in cardiac and central nervous system function. The variants identified through haplotype caller for each patient were further subject to interpretation based on ACMG/AMP standards and guidelines for the interpretation of sequence variants.^16^ This was preformed through InterVar^39^(version 0.1.7), a software program designed to assess the pathogenicity of the variants according to ACMG/AMP standards and guidelines. The pathogenicity of identified variants that were unique to SUDEP was assessed. The analytical work flow is summarized in Fig. 2. Gene expression patterns in humans for select genes were assessed by querying the EMBL-EBI Expression Atlas.^40^

### Data availability statement

All original sequencing reads has been deposited in NCBI’s public repository Sequencing Reads Archive (SRA). They are available under the BioProject ID: PRJNA436015. In particular, the bam files are available under the accession IDs: SAMN08611125, SAMN08611126, SAMN08611127, SAMN08611128, SAMN08611129,SAMN08611130, SAMN08611131, SAMN08611132, SAMN08611133, SAMN08611134,SAMN08611135, SAMN08611136, SAMN08611137, SAMN08611138 and SAMN08611139 (SRP133515). The list of figures that have raw associated data are: Fig. 3, Fig. 4, Fig. 5 (data available in Supplemental Table 2). No restrictions has been placed on data availability. Our data are published in SRA and this is the link that has our data. https://www.ncbi.nlm.nih.gov/Traces/study/?acc=SRP133515

## Electronic supplementary material

Supplemental Table 1(DOCX 57 kb)

Supplemental Table 2(XLSX 204 kb)

Supplemental Table 3(XLSX 132 kb)

Supplemental Table 4(XLSX 36 kb)

Supplemental Table 5(XLSX 15 kb)

Supplemental Table 6(XLSX 12 kb)

Supplemental Table 7(XLSX 9 kb)

Supplemental Table 8(XLSX 14 kb)

Supplemental Table 9(XLSX 8 kb)

Supplemental Figure 1(DOCX 2544 kb)

Supplemental Figure 2(DOCX 59 kb)
