# Supplementary material for: Cardiac arrhythmia and neuroexcitability gene variants in resected brain tissue from patients with sudden unexpected death in epilepsy (SUDEP)
Source: NPJ Genom Med. 2018 Mar 27;3:9. doi: 10.1038/s41525-018-0048-5 (PMC5869741; doi:10.1038/s41525-018-0048-5)
Supplement: Supplementary file 1 — Supplemental Table 1(DOCX 57 kb) [file 41525_2018_48_MOESM1_ESM.docx]

**Supplemental Table 1:** Sequencing statistics
