# Supplementary material for: Cardiac arrhythmia and neuroexcitability gene variants in resected brain tissue from patients with sudden unexpected death in epilepsy (SUDEP)
Source: NPJ Genom Med. 2018 Mar 27;3:9. doi: 10.1038/s41525-018-0048-5 (PMC5869741; doi:10.1038/s41525-018-0048-5)

**Supplemental Figure 1:** Overview of confirmed non-synonymous mutations identified in SUDEP patients (SUDEP) and Living Epilepsy patients and comparison with manually curated genes associated with epilepsy and SUDEP.


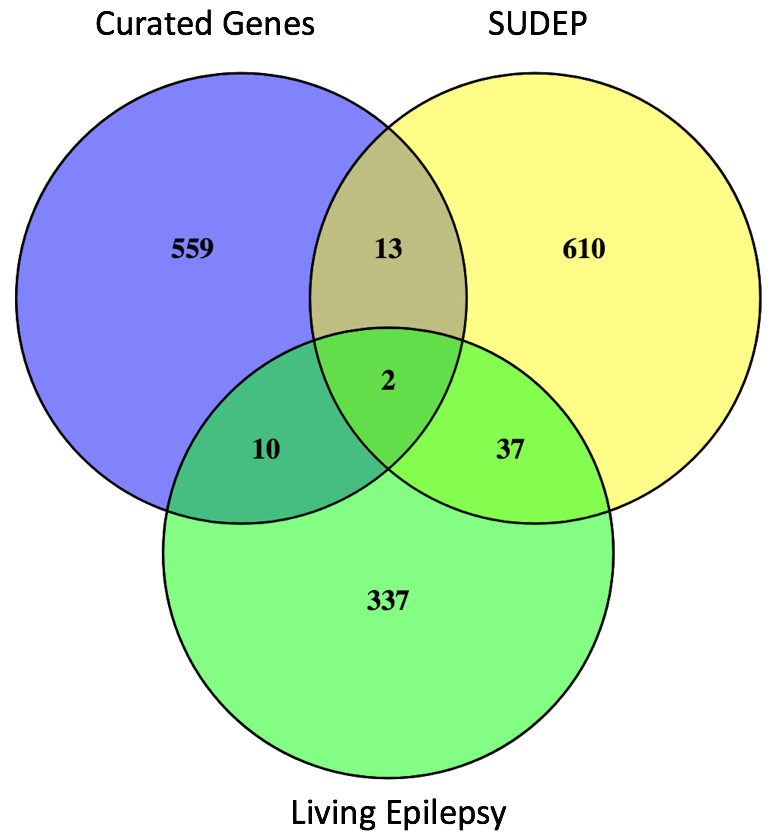

Supplement: Supplementary file 10 — Supplemental Figure 1(DOCX 2544 kb) [file 41525_2018_48_MOESM10_ESM.docx]
