# Supplementary material for: Cardiac arrhythmia and neuroexcitability gene variants in resected brain tissue from patients with sudden unexpected death in epilepsy (SUDEP)
Source: NPJ Genom Med. 2018 Mar 27;3:9. doi: 10.1038/s41525-018-0048-5 (PMC5869741; doi:10.1038/s41525-018-0048-5)
Supplement: Supplementary file 11 — Supplemental Figure 2(DOCX 59 kb) [file 41525_2018_48_MOESM11_ESM.docx]

**Supplemental Figure 2.** Plot of the number of rare variants per subject. There are more known pathogenic and likely pathogenic variants (red) in tissue from Living Epilepsy patients compared to SUDEP patients (Mann-Whitney U=9.0 , p=0.03). However, when variants of unknown significance (blue) are included, there is no difference in the burden of potentially deleterious variants between groups (Mann-Whitney U=26, p=0.89).
